# Structural Crystalline Characterization of Sakuranetin — An Antimicrobial Flavanone from Twigs of *Baccharis retusa* (Asteraceae)

**DOI:** 10.3390/molecules19067528

**Published:** 2014-06-06

**Authors:** Simone dos S. Grecco, Antônio C. Dorigueto, Iara M. Landre, Marisi G. Soares, Kevin Martho, Ricardo Lima, Renata C. Pascon, Marcelo A. Vallim, Tabata M. Capello, Paulete Romoff, Patricia Sartorelli, João Henrique G. Lago

**Affiliations:** 1 Instituto de Ciências Ambientais, Químicas e Farmacêuticas, Universidade Federal de São Paulo, 09972-270 Diadema–SP, Brazil; 2 Centro de Ciências Naturais e Humanas, Universidade Federal do ABC, 09090-400 Santo André–SP, Brazil; 3 Instituto de Química, Universidade Federal de Alfenas, 37130-000 Alfenas–MG, Brazil; 4 Escola de Engenharia, Universidade Presbiteriana Mackenzie, 01302-090 São Paulo–SP, Brazil

**Keywords:** *Baccharis retusa*, sakuranetin, X-ray diffratometry, antimicrobial activity

## Abstract

Bioactivity-guided fractionation of an antimicrobial active extract from twigs of *Baccharis retusa* C. DC. (Asteraceae) yielded the flavanone 5,4'-dihydroxy-7-methoxy-flavanone (sakuranetin) as responsible for the detected activity. The structure of the bioactive compound was established on the basis of spectroscopic data analysis, including NMR and MS. Additionally, the structure of a new crystal form of sakuranetin was confirmed by X-ray diffratometry. The minimum inhibitory concentrations (MIC) of isolated compound were determined against pathogenic yeast belonging to the genus *Candida* (six species), *Cryptococcus* (two species/four serotypes) and *S. cerevisiae* BY 4742 (S288c background) and ranged from 0.32 to 0.63 μg/μL. Our results showed that sakuranetin, which structure was fully characterized, could be used as a tool for the design of novel and more efficacious antifungal agents.

## 1. Introduction

*Baccharis* is an exclusively American genus from Asteraceae and is composed by approximately 500 species [1]. Several of these species have been used in Brazilian folk medicine as anti-inflammatory, antifungal and antimicrobial [2]. Phytochemically, this genus is known to produce different classes of natural products, including diterpenes [3,4], triterpenes [5,6], flavonoids [7,8], and trichothecenes [8]. Previously, we have investigated the composition of leaves from *B. retusa* in which were isolated several flavonoids [9], including some that displayed antiparasitic [10,11] and anti-inflammatory activities [12]. In this paper, we report the bioactivity-guided fractionation of antimicrobial extract from twigs of *B. retusa*, in order to identify the constituent(s) responsible for the detected activity. Thus, using several chromatographic procedures, was obtained the flavanone 5,4'-dihydroxy-7-methoxyflavanone (sakuranetin), which was characterized by NMR and MS analysis. Additionally, the structure of a new crystal form of sakuranetin was determined using single crystal X-ray analysis. The antimicrobial activity of the isolated compound was tested against species of pathogenic yeast belonging to the genus *Candida* (six species), *Cryptococcus*, comprising two species (four serotypes) and the laboratory standard strain *S. cerevisiae* BY 4742 (S288c background). Sakuranetin showed strong antifungal activity against these opportunistic pathogens and therefore may be considered in future studies as a prototype antifungal agent to be applied in the pharmaceutical industry.

## 2. Results and Discussion

The air-dried twigs of *Baccharis retusa*, defatted with *n*-hexane, were extracted using EtOH. The crude extract was then subjected to successive chromatographic steps using a bioactivity-guided fractionation to afford the flavanone sakuranetin as responsible for the antimicrobial activity detected in the crude extract. The structure of this compound was fully characterized using NMR and MS as well as by X-ray analysis.

### 2.1. Chemical Characterization of Sakuranetin by NMR and MS

**Figure 1:**
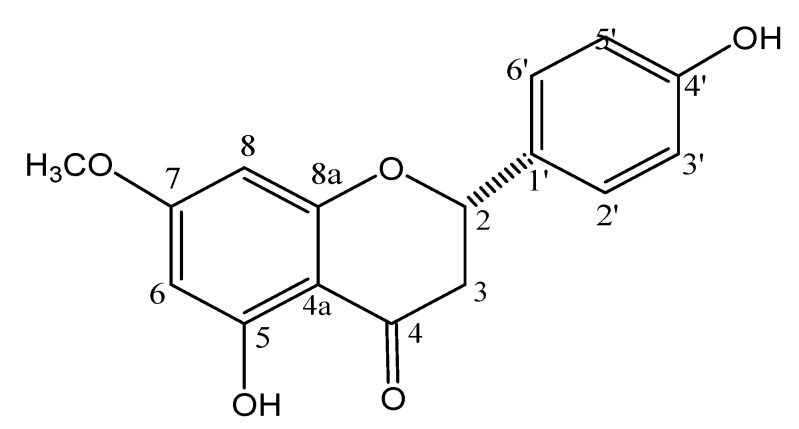
Chemical structure of 5,4'-dihydroxy-7-methoxy-flavanone (sakuranetin).

The ^1^H-NMR spectrum (CD_3_OD, 300 MHz) showed signals at δ 5.32 (dd, *J* = 13.0 and 3.0 Hz), 3.08 (dd, *J* = 17.2 and 13.0 Hz), and at δ 2.73 (dd, *J* = 17.2 and 3.0 Hz), assigned, respectively, to H-2, H-3a, and H-3b [13]. These spectra showed also doublets attributed to H-3'/H-5' at δ 6.83 (*J* = 8.5 Hz, 2H) and to H-2'/H-6' at δ 7.26 (*J* = 8.5 Hz, 2H) as well as one singlet assigned to H-6/H-8 at δ 6.01 (2H). Additionally was observed one singlet at δ 3.77 (3H), assigned to one methoxyl group linked to C-7. ^13^C and DEPT 135° NMR spectra (CD_3_OD, 75 MHz) showed peaks at range δ 94–168, which were attributed to aromatic carbons C-4a to C8a and C-1' to C-6'. Remaining signals were observed at δ 196.5 (C-4), at δ 79.1 (C-2), at δ 42.8 (C-3) and at δ 55.3 (OCH_3_). These data, associated to LRESIMS and correlations observed in the HSQC and HMBC spectra, are consistent with structure of 5,4'-dihydroxy-7-methoxyflavanone (sakuranetin-Figure 1), confirmed by comparison of spectroscopic data with those reported in the literature [11,14].

### 2.2. Structural Crystalline Characterization of Sakuranetin

**Figure 2:**
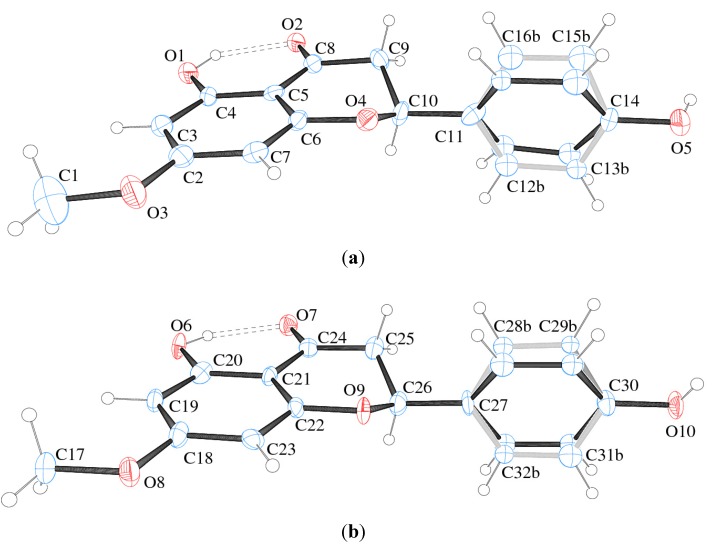
ORTEP plot and atom numbering of (**a**) molecule A and (**b**) molecule B of sakuranetin-monohydrate (S-enantiomer arbitrary chosen). Ellipsoids represent 50%-probability level. Double-dotted lines represent intramolecular H-bond. Disorder in the rings is detailed in light grey and black. Water molecules were omitted for clarity.

In the present study, a monoclinic monohydrate crystal form of sakuranetin hereafter called sakuranetin-monohydrate is reported in the space group P2_1_. The structure consists of two independent by symmetry flavanone molecules in the asymmetric unit (Z' = 2). Crystal data and structure refinement of sakuranetin are presented in Table 1. The ORTEP-3 view of the two flavanones is given in Figure 2, which shows that both molecules have disordered aromatic rings.

**Table 1:** Crystal data and structure refinement of sakuranetin.

Empirical Formula	2 (C_16_H_14_O_5_·H_2_O)
Formula weight	602.53
Temperature/K	120(1) K
Wavelength/Å	1.54180
Crystal system	Monoclinic
Space group	P2_1_
Unit cell dimensions/Å and °	*a* = 4.825(5), *b* = 13.978(5), *c* = 21.077(5)
	*β* = 91.091(5)
Volume/Å^3^	1421.3(16)
Z/Z'	4/2
Density (calculated)/Mg.m^−3^	1.422
Absorption coefficient/mm^−1^	0.921
F(000)	640
Crystal size	0.28 × 0.14 × 0.02 mm^3^
Theta range for data collection	4.20 to 63.27°
Index ranges	−5 d h d 3, −15 d k d 15, −24 d l d 23
Reflections collected	4538
Independent reflections	3241 [R(int) = 0.0269]
Completeness to theta = 63.27°	84.1%
Refinement method	Full-matrix least-squares on F^2^
Data/restraints/parameters	3241/1/395
Goodness-of-fit on F^2^	1.040
Final R index [I > 2 sigma(I)]	R1 = 0.0609, wR2 = 0.1601
R index (all data)	R1 = 0.0917, wR2 = 0.1816
Largest diff. peak and hole/e.Å^−3^	0.547 and −0.248

The model splitting homologous disordered atoms over two positions with 50% occupancy each was found to be the best one. Since there are two different molecules (due to Z' = 2) sakuranetin-monohydrate having each one two different conformer (due to disorder showed in Figure 2), the conformers will hereafter be referred to A_a_ = molecule A and conformer a; A_b_ = molecule A and conformer b; B_a_ = molecule B and conformer a; B_b_ = molecule B and conformer b.

There are three previously published crystal structures to sakuranetin. The first reported crystal structure is an anhydrous form recrystallized from synthetic product [space group P2_1_/c; cell parameters *a* = 13.172(1), *b* = 5.660(1), *c* = 18.101(2) Å, *β* = 97.28(2), *V* = 1338.6(4) Å^3^] [15]. Since the anhydrous form consists of a chiral molecule crystallized in a centrosymmetric space group, its crystal structure is an equimolar mixture of a pair of enantiomers in a well-defined arrangement (racemic crystal). This form will hereafter be referred to as anhydrous sakuranetin. Another crystal structure of a dihydrate form of sakuranetin was described [space group P2_1_2_1_2_1_; cell parameters *a* = 5.0869(10), *b* = 9.4622 (19), *c* = 32.318 (7) Å, *β* = 97.28(2), *V* = 1555.6(5) Å3] [16]. Recently it was reported a monoclinic lattice form of sakuranetin, space group P2_1_/c with *a* = 12.8531(12), *b* = 5.7141(3), *c* = 18.0355(12) Å, *β* = 97.333(8), *V* = 1313.77(16) Å^3^, *Z* = 4, M_r_ = 286.27, C_16_H_14_O_5_, *μ*(MoK*α*) = 0.108 mm^−1^, Dc = 1.447 g/cm^3^, F(000) = 600, the final R = 0.0350 and wR = 0.0859 for 2571 independent reflections (R_int_ = 0.0246) [17].

Despite not establishing the absolute configuration of the dihydrate form by the Flack methods, other authors [16] describe it as being the *S* enantiomer of sakuranetin, hereafter referred as sakuranetin-dihydrate.

**Figure 3:**
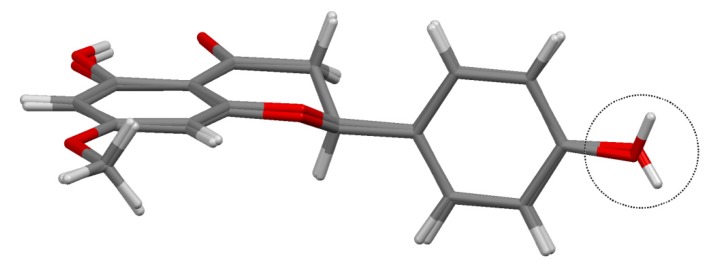
Superimposed structures for the sakuranetin-anhydrous and sakuranetin-dihydrate forms.

In Figure 3 the sakuranetin-anhydrous and sakuranetin-dihydrate structures are superimposed themselves in a capped stick fashion. The overlay of molecular backbones clearly shows the conformational similarity between homologous atoms, except to the hydroxyl hydrogen atoms of the hydroxyphenyl group, in which, the H atoms points to up and to down in sakuranetin-anhydrous and sakuranetin-dihydrate, respectively. Since there is no significant difference in terms of non-hydrogen atoms comparing the intramolecular structures of the previously determined phases of sakuranetin, hereafter sakuranetin-dihydrate will be used in the intramolecular comparisons with the four conformers determined for sakuranetin-monohydrate.

Table 2 gives the four torsion angles about the bonds by which the hydroxyphenyl group links the chromane ring. It is observed that equivalent conformers in sakuranetin-monohydrate present similar torsional angles: A_a_ ≈ B_a_ and A_b_ ≈ B_b_. As already shown in Figure 3, the intermolecular geometries of the previously determined structures are very similar, which is corroborated comparing their homologous dihedral angles (Table 2). Additionally, Table 2 shows that the conformers b (either A_b_ or B_b_) are more similar to the previously determined structures than the conformers a (either A_a_ or B_a_).

**Table 2:** Torsion angles (°) about the bonds by which the hydroxyphenyl group links the chroman rings considering all molecules as S-enantiomer.

Torsion Angle	(I)-Anhydrous ^†^	(I)-Dihydrate ^†^	(I)-MonohydrateMolecule A		(I)-MonohydrateMolecule B	
			*conformer a*	*conformer b*	*conformer a*	*conformer b*
φ1	−66.43	−66.13	−41.04	−82.46	−46.04	−78.78
φ2	111.35	112.62	137.70	106.53	126.89	100.64
φ3	56.27	56.07	85.38	43.96	76.62	43.89
φ4	−125.94	−125.19	−95.89	−127.05	−110.45	−136.69

φ1 = O4-C10-C11-C16; φ2 = O4-C10-C11-C12; φ3 = C9-C10-C11-C16; φ4 = C9-C10-C11-C12. The homologous atoms to O4, C10, C11, C12, and C16 in Molecule B are respectively O9, C26, C27, C28, and C32. **^†^** [15,16].

**Figure 4:**
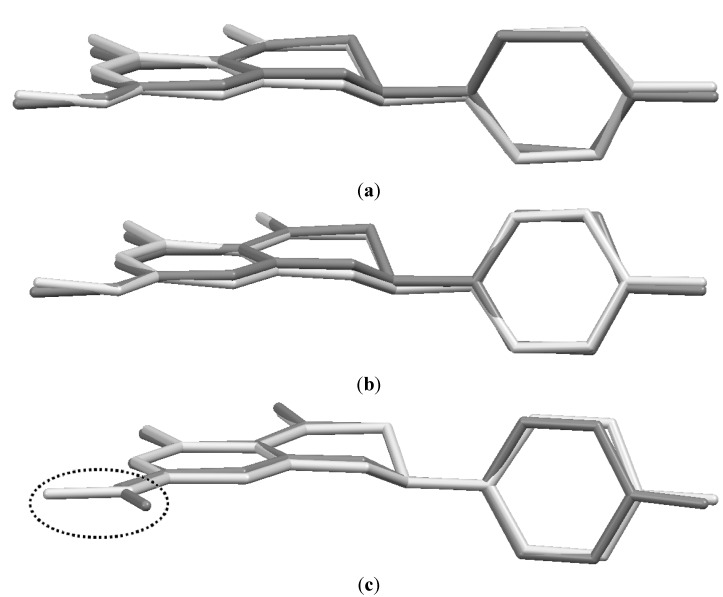
Superimposed structures for: (**a**) conformers A_a_ + B_a_ of sakuranetin-monohydrate; (**b**) conformers A_b_ + B_b_ of sakuranetin-monohydrate; and (**c**) conformers A_b_ of sakuranetin-monohydrate + sakuranetin-dihydrate. Hydrogen atoms were omitted for clarity. [Color legend: light grey = A_a_ and A_b_; dark grey = B_a_, B_b_ and sakuranetin-dihydrate].

Figure 4a,b overlay the conformers A_a_ with B_a_ and conformer A_b_ with B_b_, respectively, showing the similarity of the homologous atoms. Figure 4c overlays sakuranetin-monohydrate (conformer A_b_) with sakuranetin-dihydrate, which shows that the conformers of sakuranetin-monohydrate are geometrically more similar to the two previously determined forms.

Another intramolecular difference that deserves comment comparing sakuranetin-monohydrate with the previously reported structures is the conformation of the methoxyl groups. Figure 4c shows that the methoxyl groups have opposite geometries when sakuranetin-monohydrate and sakuranetin-dihydrate are superimposed.

The sakuranetin-monohydrate was analyzed through MOGUL [18], a knowledge base that take a molecule submitted either manually or by another computer program via an instruction-file interface and perform substructure searches of the Cambridge Structural Database (CSD) [19] for, typically, all its bond, angles and torsion angles. This study showed that the bonds C8–O2 (1.26(1) Å) and C25–O6 (1.28(1) Å) in molecules A and B respectively, are larger than expected (1.22(2) Å) when compared to similar fragments of molecules deposited in the CSD. This geometric feature is due to the presence of strong intramolecular hydrogen bonds O1–H1 O2 in Molecule A and O6–H6 O7 in Molecule B (Figure 2 and Table 3).

**Table 3:** Hydrogen bond angles (°) and distances (Å). The characters “D” and “A” refer to hydrogen bond donor and acceptor.

D–H–A	d (D–H)	d (H–A)	d (D–A)	<(DHA)
O1-H1...O2	0.82	1.90	2.632(9)	147.7
O6-H6...O7	0.82	1.89	2.622(9)	148.8
O5-H5...O12 ^i^	0.82	2.18	2.97(1)	161.5
O10-H10a...O11 ^iv^	0.82	2.18	2.97(1)	161.5
O12-H12c...O1	1.056(6)	1.783(6)	2.839(9)	179.5(5)
O12-H12d...O5 ^iv^	0.993(6)	1.632(7)	2.625(9)	179.6(5)
O11-H11b...O6	1.096(5)	1.830(6)	2.926(8)	179.6(4)
O11-H11a...O10 ^i^	1.105(6)	1.826(7)	2.93(1)	179.9(4)
O1-H1...O7 ^vii^	0.82	2.41	2.87(1)	116.3
O6 ^vii^-H6...O2	0.82	2.41	2.87(1)	116.3

**Figure 5:**
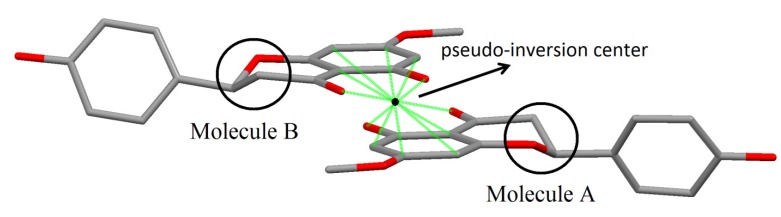
Details of molecules A and B in sakuranetin-monohydrate related to the pseudo-inversion center. Solid green lines show atoms related by the inversion center (black point). The inversion symmetry is forbidden for the stereogenic atoms C10 and C26 in the homologous chromane rings (highlighted by circles).

**Figure 6:**
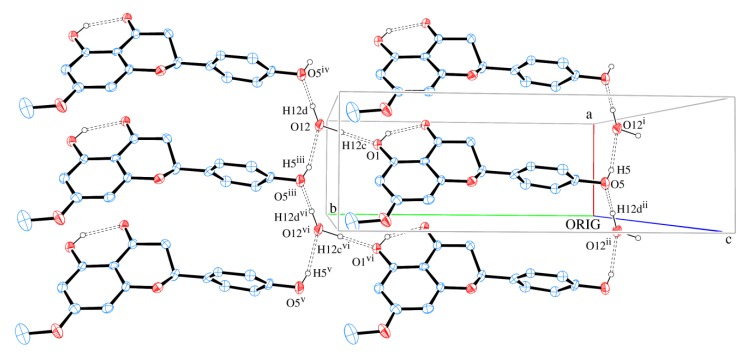
A section of the supramolecular 2D assembly of sakuranetin-monohydrate (Molecule A), projected onto (001). The chains propagation along [100] and [010] are evident. Hydrogen bonds are shown as dashed lines. Displacement ellipsoids are drawn at the 30% probability level. H of carbon atoms were omitted for clarity. [Symmetry codes: (i) x, y − 1, z; (ii) −x, −y, z; (iii) x, y + 1, z; (iv) x + 1, y + 1, z; (v) x − 1, y + 1, z; (vi) x − 1, y, z].

Symmetry transformations used to generate equivalent atoms (as Figure 5 and Figure 6): (i) x, y − 1, z; (iv) x + 1,y + 1, z; (vii) x + 1, y, z.

Taking in account the whole geometry (intra and intermolecular structures) of sakuranetin-monohydrated, it is clearly observed a pseudo-symmetry phenomenon. This effect is not uncommon, being 27% of the structures with Z' > 1 showing approximate symmetry elements [20]. The statistical analysis and systematic absences indicate two possible space groups with 2 mL aue symmetry, P2_1_ or P2_1_/m, with 83.1% of certain for the later. However, it was impossible to solve a reasonable structure in the centrosymmetric space group P2_1_/m and then P2_1_ was tried giving a satisfactory structure determination. In fact, examining the two crystallographically independent molecules in the asymmetric unit solved in P2_1_ space group, was observed that they are related by a pseudo-inversion center, *i.e.*, only part of the two individuals molecules are related by inversion center (Figure 5). Indeed, a centrosymmetric space group is impossible because the isolated chiral molecule is enantiopure/homochiral.

The packing of sakuranetin-monohydrate phase is mainly stabilized by intermolecular classical hydrogen bonds. Figure 6 shows 2D networks parallel to ab plane formed by A molecules. In this layer, the oxygen atom (O12) of molecule of water acts as H bond donor to O1 and O5 (from hydroxyl groups) and also as H bond acceptor from O5 [see symmetry codes in Figure 5 and Table 2], forming a supramolecular structure with 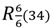 assemblies [21].

**Figure 7:**
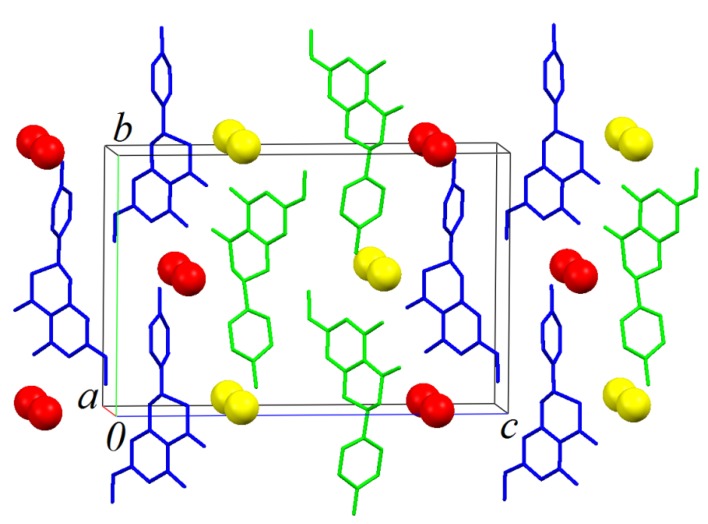
The crystal packing illustration of sakuranetin-monohydrate onto the *bc* plane.

**Figure 8:**
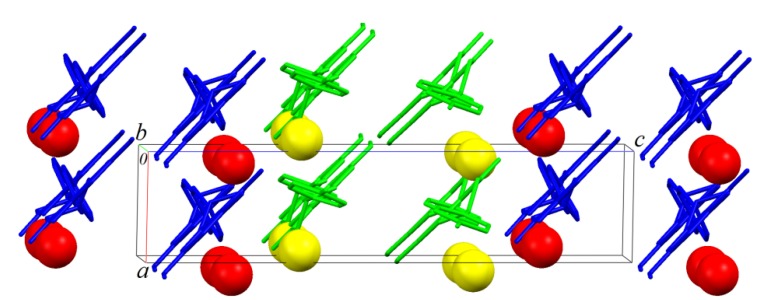
The crystal packing illustration of sakuranetin-monohydrate onto the *ac* plane.

Similar layers also parallel to *ab* plane are formed by B molecules. An AABBAA fashion staking is formed along [001] (Figure 7 and Figure 8).

**Figure 9:**
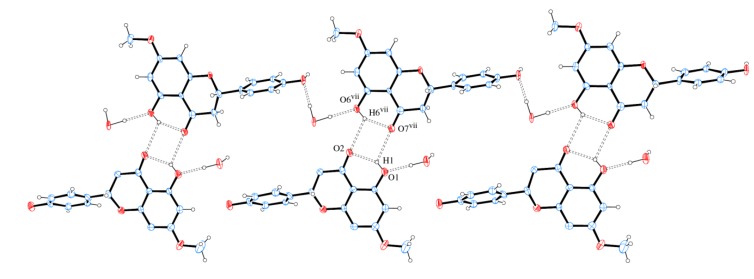
A section of the supramolecular double chain of sakuranetin-monohydrate projected onto (104) with double chain propagation along [010]. Hydrogen bonds are shown as dashed lines. Displacement ellipsoids are drawn at the 30% probability level. H of carbon atoms were omitted for clarity. [Symmetry code: (vii) x + 1, y, z].

Figure 9 shows that neighbor layers AB (or BA) are themselves linked by intermolecular H bonds. It is observed that O1 is H bond donor either to O2 (intra) or O7 (intermolecular), whereas O6 is H bond donor either to O7 (intra) or O2 (intermolecular). A square-like ring with an 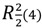 assembly is formed containing two bifurcated H bonds. The hydrogen bonds geometries are given in Table 2.

As observed in Figure 8, which shows the packing of the monohydrate phase onto plane _bc_, that green (Molecules A) and blue (Molecules B) chains formed along [10] are, as expected, related to the 2_1_-fold screw axis parallel to unit cell b axis of the P2_1_ space group. Is it interesting to note that green and blue chains are apparently related to a glide plane (glide c) normal to the unit cell b axis. This is of course a not real crystallographic symmetry because the homochirality discussed above, but shows that the packing analysis point out to a pseudo-symmetry closer to P2_1_/c than P2_1_/m suggested to the systematic absences analysis.

### 2.3. Antifungal Sensibility Test

In a preliminary screen, crude extract, fractions A–F and B1–B7 (see Experimental) were tested by disk diffusion assay, using fluconazole (100 µg) as positive and sterile water as negative controls. Crude extract, fractions B and B3 produced growth inhibition at 100 µg per disk for all tested microorganisms. In order to determine the antimicrobial activity of sakuranetin, isolated from bioactive fraction B3, the minimum inhibitory concentrations (MIC-concentration range: 0.003–1.0 μg/μL) were determined. As showed at Table 4, sakuranetin exhibited between 98% and 99% of growth inhibition at 0.63 µg/µL to all tested *Candida* strains, except for *C. albicans*, which was more sensitive to sakuranetin at 0.32 µg/µL (99% inhibition). *Cryptococcus* species displayed similar behavior: *C. neoformans* serotype A (var. *grubbii*) strain and *C. gatti* (R265) were inhibited at 99% and 97% at 0.32 µg/µL of sakuranetin, respectively. These two strains maintained an inhibition rate reaching 15% and 17% growth inhibition at 0.04 µg/µL, indicative of moderate growth. This result suggests they are more sensitive than other *Cryptococcus* and *Candida* strains. *C. gatti* (NIH 312) and *S. cerevisiae* showed 97% and 99% inhibition at 0.32 µg/µL, respectively, which is similar to what was detected for *C. albicans*. The most sensitive strains was *C. neoformans* serotype D (JEC21) which had 98% inhibition at 0.08 µg/µL. *C. neoformans* serotype A and D are the most clinical relevant types, therefore, these results will be important regarding future treatment.

**Table 4:** Target strains used for antifungal activity assays and MIC resultsto sakuranetin.

Species	Designation	Source *	MIC (µg/µL) Sakuranetin **
*Candida dubliniensis*	ATCC 7978	ATCC	0.63 (99%)
*Candida tropicalis*	ATCC 13803	ATCC	0.63 (98%)
*Candida glabrata*	ATCC 90030	ATCC	0.63 (99%)
*Candida parapsilosis*	Clinical isolate 68	CBMAI	0.63 (98%)
*Candida krusei*	Clinical isolate 9602	CBMAI	0.63 (99%)
*Candida albicans*	CBMAI 560	CBMAI	0.32 (99%)
*Cryptococcus neoformans*	KN99α (serotype A)	LIMIc	0.32 (99%)
*Cryptococcus gattii*	R265 (serotype B)	LIMIc	0.32 (97%)
*Cryptococcus gattii*	NIH312 (serotype C)	LIMIc	0.32 (97%)
*Cryptococcus neoformans*	JEC21 (serotype D)	LIMIc	0.08 (98%)
*Saccharomyces cerevisiae*	BY4742	LIMIc	0.32 (99%)

* ATCC: American Type Culture Collection; CBMAI: *Centro Brasileiro de Microrganismos Ambientais e Industriais*; LIMic collection: *Laboratório de Interações Microbianas-UNIFESP*. ** Percentages in parenthesis represent the growth inhibition compared to the no drug control fluconazole (100% growth).

The antifungal activity of sakuranetin was previously reported against phytopathogenic *Cladosporium* sp. [22] and against clinical strains of *Trichophyton rubrum* and *T. mentagrophytes* [23]. In this work, purified sakuranetin displayed also a good potential as antifungal agents for opportunistic pathogenic yeasts. These results indicated that sakuranetin could be used as a tool for the design of novel and more efficacious antifungal agents.

## 3. Experimental

### 3.1. General Experimental Procedures

^1^H, ^13^C, DEPT 135°, HSQC, and HMBC NMR spectra were recorded at 300 MHz (^1^H nucleus) and 75 MHz (^13^C nucleus) in a Bruker Ultrashield 300 Advance III spectrometer using CD_3_OD (Aldrich) as solvent and internal standard. Chemical shifts are reported in δ units (ppm) and coupling constants (*J*) in Hz. LRESIMS was measured in Micromass Platform mass spectrometer, operating at positive mode. Flash silica gel (Merck, 230–400 mesh) and Sephadex LH-20 (Aldrich) were used for column chromatographic separation, while silica gel 60 F_254_ (Merck) was used for analytical (0.25 mm) and preparative (1.00 mm) TLC. Crude extract from twigs of *B. retusa* was prepared using a Dionex 350 Accelerated Solvent Extraction (ASE) at room temperature.

### 3.2. Plant Material

*Baccharis retusa* DC. twigs were collected in Campos do Jordão, SP, Brazil, in October 2010 and were identified by Oriana A. Fávero. Voucher specimen has been deposited at Herbarium of Instituto de Botânica-SEMA, São Paulo, SP, Brazil.

### 3.3. Extraction and Isolation

Dried and powdered twigs of *B. retusa* (150 g) were exhaustively defatted with n-hexane than extracted with EtOH affording 7 g of a syrupy extract, after solvent evaporation under reduced pressure. Part of this extract (4 g) was subjected to flash column chromatography (silica gel) eluted with increasing amounts of MeOH in CH_2_Cl_2_ to give six fractions of 200 mL each: A (95% of CH_2_Cl_2_, 114 mg), B (90% of CH_2_Cl_2_, 844 mg), C (80% of CH_2_Cl_2_, 325 mg), D (70% of CH_2_Cl_2_, 547 mg), E (50% of CH_2_Cl_2_, 663 mg), and F (100% of MeOH, 448 mg), being the antimicrobial activity detected in fraction B. Part of this material (500 mg) was subjected to Sephadex LH-20 column chromatography eluted with CHCl_3_:MeOH 1:1. This procedure afforded seven fractions (B1–B7), in which antimicrobial activity was detected at B3 (214 mg). This fraction was than purified by prep. TLC (silica gel) eluted with CH_2_Cl_2_/MeOH 95:5 to afford 93 mg of sakuranetin.

### 3.4. 5,4'-Dihydroxy-7-methoxyflavanone (sakuranetin)

^1^H-NMR (300 MHz, CD_3_OD) δ_H_: 7.26 (d, *J* = 8.5 Hz, H-2'/H-6'), 6.83 (d, *J* = 8.5 Hz, H-3'/H-5'), 6.01 (s, H-6/H-8), 5.32 (dd, *J* = 13.0 and 3.0 Hz, H-2), 3.77 (s, OCH_3_-7), 3.08 (dd, *J* = 17.2 and 13.0 Hz, H-3a), 2.73 (dd, *J* = 17.2 and 3.0 Hz, H-3b). ^13^C-NMR (75 MHz, CD_3_OD) δ_C_: 196.5 (C-4), 168.0 (C-4'), 163.6 (C-7), 163.0 (C-5), 157.4 (C-8a), 129.0 (C-1'), 127.7 (C-2'/C-6'), 127.6 (C-6), 115.3 (C-3'/C-5'), 102.8 (C-4a), 93.9 (C-8), 79.1 (C-2), 55.3 (OCH_3_), 42.8 (C-3). LRESIMS *m/z* 287 [M+H]^+^.

### 3.5. X-ray Crystallography

Colorless plate-like crystals of sakuranetin were obtained by slow evaporation of MeOH. A well-shaped and suitably-sized single crystal was selected for the X-ray diffraction structure determination experiment. The X-ray intensity data were collected at 120 K on a Gemini-Oxford Diffractometer, using MoKα graphite monochromated radiation. The programs CrysAlis CCD and CrysAlis RED [24] were used for data collection, cell refinement and data reduction. The structure was solved by direct methods using the software Sir-92 [25] and the refinement was carried out using SHELXL-2013 [26]. The carbon and oxygen atoms of the molecule were clearly solved and full matrix least-squares refinement of these atoms with anisotropic thermal parameters was carried on, except for the disordered C atoms that were refined with isotropic thermal parameters. Hydrogen atoms on carbon and oxygen atoms were positioned stereochemically and were refined with fixed individual displacement parameters [Uiso (H) = 1.5 Ueq (C or O) for methyl and hydroxyl groups or 1.2 Ueq (C) for methine and methylene groups] using a riding and rotating group model (for methyl and hydroxyl groups), with C-H bond lengths of 0.96, 0.97, 0.98 and 0.82 Å for methyl, methine, methylene and hydroxyl groups, respectively. The hydrogen atoms from water molecule were located by difference Fourier synthesis and were kept as rigid group together with its respective oxygen atoms during the subsequent refinements. WINGX software was used to analyze and prepare the data for publication [27]. Molecular graphics were prepared using ORTEP-3 for Windows [28] and Mercury [29]. Since the most electron-rich atom is the oxygen atom, which does not have an anomalous scattering large enough (using MoKa radiation), it was not possible to determine the enantiomer present using the Flack method [30]. In this way, the S-enantiomer was chosen during the crystal structure determination/refinement using the same absolute structure determined for sakuranetin previously described in literature [16,31,32]. Crystallographic data for the structural analysis of the compound discussed here has been deposited at the Cambridge Crystallographic Data Centre as a supplementary publication under number CCDC 998334. Copies of the data can be obtained, free of charge, on application to CCDC, 12 Union Road, Cambridge CB12 1EZ, UK [fax: +44 1223 336033 or e-mail: deposit@ccdc.cam.ac.uk].

### 3.6. Disk Diffusion Assay

Antimicrobial activity was initially evaluated by the disk diffusion method according to CLSI (M2-A8) former National Committee for Clinical Laboratory Standards (NCCLS) with the following modifications. In brief, thin agar plates were prepared with 10 mL of yeast medium YEPD. Three milliliters of liquid cultures were grown at 30 °C with aeration (150 rpm) overnight on YEPD and a top agar was prepared by mixing 100 µL of each culture with 10 mL of soft agar medium for confluent plates (YEPD) and poured on top of the thin agar (2% agar). Sterilized 6 mm filter paper disks were then impregnated with 5 µL of extracts, fractions or sakuranetin. The disks were placed on top of agar plates and incubated at 30 °C for 24 or 48 h. Fluconazole was used as positive control and negative control was prepared by impregnating the paper disks with the same amount of DMSO used to dilute the samples. All tests were performed in triplicate. The inhibition zone (IZ) was determined by measuring the whole halo diameter divided by the disk size (6 mm) [33].

### 3.7. Minimum Inhibitory Concentration

Microdilution tests were conducted in sterile 96 well micro titer plates in a total volume of 100 µL according to the CLSI Protocol (M27–A2) former National Committee for Clinical Laboratory Standards (NCCLS), with the following modifications: microorganisms were cultured in test tubes overnight at 30 °C in 3 mL medium YEPD in a rotary shaker (150 rpm). The cultures were diluted and adjusted to (1–2) × 10^2^ CFU/mL, which was confirmed by viability counts on YEPD plates (100 µL of diluted cells). Samples diluted in DMSO were serial diluted two-fold and tested. A sterilization control containing medium only (negative control) and growth control containing cell and DMSO (1%) were included as controls. Micro titer plates were then incubated at 30 °C for 24 or 48 h. Finally, the absorbance at 600 nm was measured in a plate reader (MT-960, Logen) and the minimum inhibitory concentration was considered the lowest concentration at which inhibition was at least 90% of the no drug control and visual growth inhibition was detected. All wells were stained with the vital staining rezasurin (0.005% in sterile PBS buffer) in order to improve visual inspection. All tests were performed in duplicates [33,34].

## 4. Conclusions

In conclusion, the flavanone 5,4'-dihydroxy-7-methoxyflavanone (sakuranetin) was isolated from twigs of *B. retusa* and identified by comparison of their NMR and MS data with those reported in the literature. In addition, the stereochemistry and crystal packing of sakuranetin was elucidated by single crystal X-ray diffratometry. This compound was also evaluated for its inhibitory activity against pathogenic yeast belonging to the genus *Candida*, *Cryptococcus* and *S. cerevisiae*. The obtained data showed that sakuranetin exhibited the strongest antimicrobial activity (MIC values ranging from 0.32 to 0.63 μg/μL) suggesting that this compound could be used as scaffolds for the design of novel antifungal derivatives.
